# 基于沸石咪唑酯骨架复合微球的分散固相萃取-气相色谱-质谱法检测牛奶中18种多氯联苯

**DOI:** 10.3724/SP.J.1123.2024.07005

**Published:** 2025-06-08

**Authors:** Wending NIE, Sijie SHUAI, Ke HU, Xiaolei CUI, Tengfei LI

**Affiliations:** 1.河北工程大学生命科学与食品工程学院，河北 邯郸 056038; 1. School of Life Sciences and Food Engineering，Hebei University of Engineering，Handan 056038，China; 2.河北省地质矿产勘查开发局 第一地质大队实验室，河北 邯郸 056000; 2. Laboratory of The First Geological Team，Hebei Bureau of Geology and Mineral Resources Exploration and Development，Handan 056000，China

**Keywords:** 沸石咪唑酯骨架, 壳聚糖, 分散固相萃取, 气相色谱-质谱, 多氯联苯, 牛奶, zeolitic imidazolate frameworks （ZIFs）, chitosan （CS）, dispersive solid-phase extraction （DSPE）, gas chromatography-mass spectrometry （GC-MS）, polychlorinated biphenyls （PCBs）, milk

## Abstract

多氯联苯（PCBs）是一类持久性有机污染物，尽管在全球范围内已被禁用，但仍以痕量水平存在于食品和环境中。PCBs残留会对人类健康和生态环境造成严重威胁，因此建立可靠的PCBs富集检测方法具有重要意义。本文以原位合成法制备壳聚糖/沸石咪唑酯骨架复合微球（CS@ZIF-8）作为分散固相萃取（DSPE）吸附剂，结合气相色谱-质谱（GC-MS），建立了一种测定牛奶中18种PCBs的分析方法。通过扫描电子显微镜、傅里叶红外光谱、X射线衍射和氮气吸附/脱附对制备的材料进行表征。考察了吸附剂用量、萃取时间、解吸时间和解吸溶剂等因素对萃取效率的影响，得到最佳萃取条件：20 mg CS@ZIF-8作为吸附剂，振荡提取30 min，1 mL正己烷超声解吸8 min。在最佳萃取条件下，18种PCBs在1~200 μg/L范围内具有良好的线性关系，相关系数（*r* ^2^）均大于0.999，检出限（*S/N*=3）为0.06~0.24 μg/L，定量限（*S/N*=10）为0.19~0.79 μg/L，日内和日间精密度（*n*=6）分别为2.5%~5.3%和4.3%~5.9%，不同批次材料间精密度（*n*=3）为4.9%~9.7%。选择全脂牛奶和脱脂牛奶对本方法的适用性进行考察，在5、20和100 μg/L 3个水平下，18种PCBs的加标回收率为84.8%~114.3%。考察了CS@ZIF-8的重复利用性，经过4次吸附-解吸循环后，加标回收率仍能达到70%以上。本方法操作简便，萃取时间短，准确度高，为牛奶样品中PCBs的高效检测提供了有力支持。

## Abstract (en)

Polychlorinated biphenyls （PCBs） are hazardous， persistent organic pollutants that are widely used industrially. Although the use of PCBs is banned in many countries， they are still present at trace levels in food and the environment. PCBs are highly chemically stable and lipophilic； hence， they are easily enriched and accumulate in the human body through milk and dairy products. PCBs residues pose serious threats to human health； therefore establishing a reliable enrichment method is an important objective. Sample pretreatment is required to efficiently extract target PCBs owing to sample-matrix complexity and their low contents. Efficient adsorbents form the cores of novel sample-pretreatment technologies， and designing new stable adsorbents is crucial for the further development of pretreatment techniques. Zeolitic imidazolate frameworks （ZIFs） are a family of metal-organic frameworks composed of imidazole linkers and metal ions. Their large surface areas， good stabilities， high porosities， and ease of modification are distinct advantages； consequently， ZIFs are widely used to adsorb organic pollutants. However， powdered ZIFs are difficult to separate and collect， which provides reuse challenges； hence， preparing ZIF composites with other functional materials is a highly effective way of addressing this challenge. Chitosan （CS） is an inexpensive and biodegradable natural polysaccharide that gelates easily. The structure of CS contains many free amino and hydroxyl groups that facilitate chemical modification and hybridization； consequently， CS is a matrix commonly used in composite materials. In this study， we prepared CS@ZIF-8 composite beads by the in-situ synthesis of ZIF-8 on chitosan through acid-solubilization/base-fixation. An analytical method for determining 18 PCBs in milk was developed using CS@ZIF-8 composite microspheres as the adsorbent for dispersive solid-phase extraction （DSPE） coupled with gas chromatography-mass spectrometry （GC-MS）.The CS@ZIF-8 composite microspheres were characterized by scanning electron microscopy （SEM）， Fourier-transform infrared （FT-IR） spectroscopy， X-ray diffractometry （XRD）， and nitrogen-adsorption-desorption experiments， which confirmed that the material had been successfully prepared. How adsorbent dosage， extraction and desorption times， and type and volume of the desorption solvent affect the extraction efficiency were investigated， with the following optimal extraction conditions determined： 20 mg of CS@ZIF-8 as the adsorbent， 30 min of extraction by shaking， and 8 min of ultrasonic desorption with 1 mL of *n*-hexane. The 18 PCBs exhibited good linearities in the 1–200 μg/L under these optimal conditions， with coefficients of determination （*r* ^2^） exceeding 0.999. Detection limits （*S/N*=3） ranged between 0.06 and 0.24 μg/L， with quantification limits （*S/N*=10） of 0.19–0.79 μg/L. Repeatability experiments were performed by the addition of 100 μg/L of the 18 PCBs， which exhibited intra-day and inter-day precisions （*n*=6） of 2.5%–5.3% and 4.3%–5.9%， respectively， while inter-batch material precisions （*n*=3） ranged between 4.9% and 9.7%. The applicability of the developed method was investigated by selecting whole milk and skim milk as samples based on PCBs lipophilicity. Spiked recovery experiments were conducted at three concentrations （5， 20， and 100 μg/L）， with the 18 PCBs exhibiting spiked recoveries of 84.8%–114.3%. CS@ZIF-8 not only has a larger specific surface area than CS， but it also adsorbs PCBs through *π*-*π* interactions and hydrophobicity， leading to superior extraction efficiency. CS@ZIF-8 exhibited spiked recoveries exceeding 70% for all samples after four adsorption-desorption cycles during reproducibility testing. The developed method provides a simplified extraction process by eliminating the need for centrifugation or filtration steps that are usually associated with conventional DSPE. In addition， the developed method is highly sensitive， precise， and accurate， with adsorbent reusability a noteworthy feature， thereby supporting the simple and efficient detection of PCBs in milk samples.

多氯联苯（polychlorinated biphenyls，PCBs）是一类氯代芳香族化合物，由于出色的稳定性和电绝缘性，曾被广泛用于阻燃剂、热载体和绝缘油等工业产品^［1，2］^。PCBs是《斯德哥尔摩公约》中优先控制的12类持久性有机污染物之一，具有生物蓄积性、高脂溶性、三致性和远距离迁移性，能够通过食物链的生物放大效应对人类健康和生态系统造成严重威胁^［3，4］^。自20世纪70年代以来，全球多个国家已禁止使用PCBs，但它们仍微量存在于食品和环境中^［5］^。PCBs因脂溶性强而易在牛奶等乳制品中积累。已有多项研究报道了牛奶中检出PCBs的情况，例如Lu等^［6］^对15份牛奶样品中5种PCBs含量进行测定，在10份牛奶样品中检出PCBs；喻德忠等^［7］^对30份牛奶样品中6种PCBs进行测定，30份样品均有PCBs检出，6种PCBs总含量为107.2~4 311.2 pg/g脂重。张瑞等^［8］^对9类总膳食样品中7种PCBs进行检测，其中乳类PCBs总含量为30.33 pg/g湿重。牛奶作为日常饮食的重要部分，其安全性直接关系到公众健康。因此，开发一种高效、准确检测PCBs的方法十分必要。

气相色谱-质谱（GC-MS）因灵敏度高、定性定量准确成为PCBs的常用分析方法^［9］^。但样品基质复杂且PCBs含量低，难以直接分析，需要对样品进行预处理。传统的样品预处理方法如索氏提取^［10］^、液液萃取^［11］^和加速溶剂萃取^［12］^等都存在有机溶剂消耗大、耗时长等问题。近年来，新型样品预处理技术如固相萃取（SPE）^［13］^、固相微萃取（SPME）^［14］^、磁固相萃取（MSPE）^［15］^和分散固相萃取（dispersive solid-phase extraction，DSPE）^［6，16，17］^等得到了迅速发展。其中DSPE试剂用量少、成本低和重现性好，已被广泛用于样品预处理^［18］^。然而DSPE常规吸附剂为粉末，分离时需要离心或过滤，操作复杂耗时，还可能因为分离不完全污染仪器。因此，制备高效的定型吸附剂（膜、纤维、球体等）已成为DSPE的研究热点。例如，Fan等^［19］^利用共价有机骨架改性泡沫镍（NF@COF）作为DSPE吸附剂，成功检测了中草药中16种多环芳烃。使用镊子快速分离吸附剂，使得萃取过程更加高效环保。Zhang等^［20］^采用溶胶-凝胶法合成Uio-66-PDMS微珠，直接提取均质柑橘样品中的植物生长调节剂，有效去除了基质干扰，简化了前处理流程。

目前，制备定型吸附剂的材料有泡沫镍^［19， 21］^、三聚氰胺海绵^［22］^和壳聚糖（chitosan，CS）^［23］^等。CS是一种成本低、可生物降解和易凝胶化的天然多糖，因其表面含有丰富的-OH和-NH_2_基团，通常被用作复合材料的基材^［24，25］^。沸石咪唑酯骨架（zeolitic imidazolate frameworks， ZIFs）是由咪唑连接体和金属离子组成的一类金属有机骨架，具有比表面积大、稳定性好、孔隙率高和易于修饰等优点^［26］^。ZIF-8作为ZIFs材料的典型代表之一，由锌离子和2-甲基咪唑自组装而成，因疏水性强和拥有*π*电子共轭体系常被用于有机污染物吸附^［27］^。因此，基于二者优势可将ZIF-8与CS复合，制备一种可塑性强、吸附容量大的复合吸附剂用于预处理PCBs。本工作以CS为载体，采用酸溶解/碱固定的方法原位合成ZIF-8，制备了CS@ZIF-8复合微球，并将其用于DSPE，结合GC-MS建立了一种检测牛奶中18种PCBs的DSPE-GC-MS方法（见图1）。本方法不需要常规的离心或过滤操作，为PCBs的简便、低成本和高效检测提供了新思路。

**图1:**
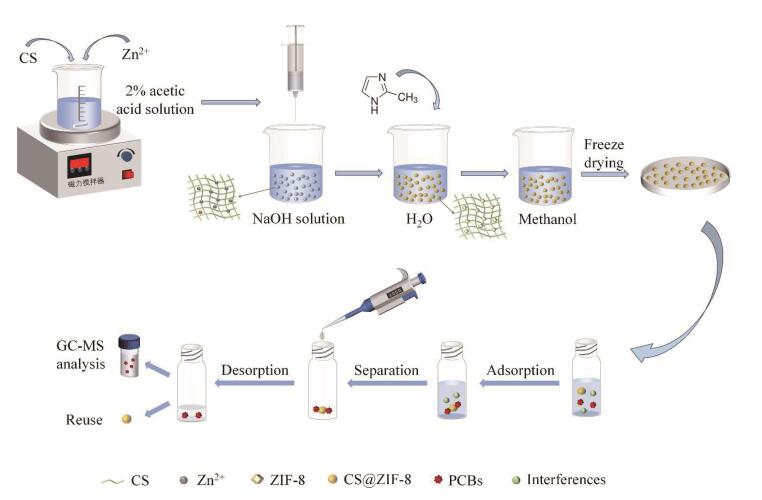
CS@ZIF-8制备和DSPE流程示意图

## 1 实验部分

### 1.1 仪器与试剂

8890-5977B气相色谱-质谱联用仪（美国安捷伦公司）；D8-ADVANCE X射线衍射仪（德国布鲁克公司）；Hitachi S-4800场发射扫描电子显微镜（日本日立公司）；ASAP 2460全自动比表面积及孔径分析仪（美国麦克公司）；Nicolet 6700傅里叶红外光谱仪（美国尼高力公司）；LGJ-18S型真空冷冻干燥机（北京松源华兴科技发展有限公司）；SP200-2T多通道磁力搅拌器（杭州米欧仪器有限公司）。

18种PCBs混合标准溶液（100 mg/L，正己烷）、正己烷、二氯甲烷、甲苯、乙酸乙酯、丙酮、甲醇、乙腈（色谱纯）均购于上海安谱实验科技股份有限公司。冰乙酸、氢氧化钠（分析纯）购于上海国药集团化学试剂有限公司，六水合硝酸锌（Zn（NO_3_）_2_·6H_2_O，分析纯）购于天津市风船化学试剂科技有限公司，二甲基咪唑（分析纯）购于上海阿拉丁化学试剂有限公司，CS（脱乙酰度≥95%，黏度100~200 mPa·s）购于上海易恩化学技术有限公司。

### 1.2 气相色谱-质谱条件

#### 1.2.1 色谱条件

色谱柱：HP-5 MS 毛细管柱（30 m×0.25 mm×0.25 μm，美国安捷伦公司）； 载气：高纯氦气（纯度≥99.999%）；流速：1.0 mL/min；进样量：1 µL；进样口温度：270 ℃，采用不分流进样模式；升温程序：初始温度80 ℃，以 20 ℃/min 速率升至 180 ℃，然后以5 ℃/min 速率升温到 270 ℃，最后以 20 ℃/min 速率升温到 300 ℃，保持 3 min。

#### 1.2.2 质谱条件

离子源：电子轰击源（EI）；电离电压：70 eV；四极杆温度：150 ℃；离子源温度：230 ℃；传输线温度：280 ℃；溶剂延迟：9.5 min；选择离子监测（SIM）模式采集。18种PCBs的保留时间、定量和定性离子等参数见表1。

**表 1:** 18种PCBs的保留时间和质谱参数

No.	Analyte	Retention time/min	Quantitative ion （*m/z*）	Qualitative ions （*m/z*）
1	PCB28 （2，4，4′-三氯联苯）	9.862	256	186，258
2	PCB52 （2，2′，5，5'-四氯联苯）	10.730	292	220，290
3	PCB101 （2，2′，4，5，5′-五氯联苯）	13.194	326	256，328
4	PCB81 （3，4，4′，5-四氯联苯）	14.023	292	220，290
5	PCB77 （3，3′，4，4′-四氯联苯）	14.328	292	220，290
6	PCB123 （2′，3，4，4′，5-五氯联苯）	15.046	326	254，328
7	PCB118 （2，3′，4，4′，5-五氯联苯）	15.121	326	254，328
8	PCB114 （2，3，4，4′，5-五氯联苯）	15.474	326	254，328
9	PCB153 （2，2′，4，4′，5，5′-六氯联苯）	15.829	360	290，362
10	PCB105 （2，3，3′，4，4′-五氯联苯）	15.962	326	254，328
11	PCB138 （2，2′，3，4，4′，5′-六氯联苯）	16.722	360	290，362
12	PCB126 （3，3′，4，4′，5-五氯联苯）	17.016	326	256，328
13	PCB167 （2，3′，4，4′，5，5′-六氯联苯）	17.634	360	290，362
14	PCB156 （2，3，3′，4，4′，5-六氯联苯）	18.381	360	290，362
15	PCB157 （2，3，3′，4，4′，5′-六氯联苯）	18.563	360	290，362
16	PCB180 （2，2′，3，4，4′，5，5′-七氯联苯）	18.922	394	324，396
17	PCB169 （3，3′，4，4′，5，5′-六氯联苯）	19.615	360	290，362
18	PCB189 （2，3，3′，4，4′，5，5′-七氯联苯）	20.836	394	324，396

### 1.3 标准溶液配制

用正己烷配制质量浓度为10 mg/L的PCBs混合标准溶液，于4 ℃冰箱冷藏保存。后续按照实验要求稀释成所需浓度的PCBs系列标准工作溶液。

### 1.4 CS@ZIF-8复合微球的制备

参考文献［^28^］的方法，将0.48 g CS溶解在16 mL 2%（v/v）乙酸水溶液中，然后将1.78 g Zn（NO_3_）_2_·6H_2_O加入到上述溶液中，室温下磁力搅拌3 h获得均匀分散体。用注射器将混合溶液滴入100 mL氢氧化钠（l mol/L）溶液中，将所得微球用超纯水洗涤数次直至洗脱液呈中性，然后浸入40 mL含3.94 g 2-甲基咪唑的水溶液中，室温下放置24 h。将获得的产物用甲醇洗涤3次并在甲醇中浸泡24 h。冷冻干燥后得到CS@ZIF-8复合微球。

### 1.5 牛奶样品的采集和处理

牛奶样品购于邯郸本地市场。准确移取1 mL牛奶样品于10 mL 离心管中，加入3 mL乙腈涡旋2 min。然后以8 000 r/min离心5 min，将上清液转移至浓缩杯中。重复浸提步骤两次，合并浸提液，氮吹去除乙腈，用超纯水稀释至10 mL，将得到的样品溶液转移到50 mL离心管中。

向10 mL样品溶液中加入 20 mg CS@ZIF-8吸附剂，将混合物振荡提取30 min以达到吸附平衡。用移液枪移取样品溶液，并将其弃去。然后向离心管中加入 1 mL正己烷超声解吸 8 min。移取洗脱液过 0.22 μm有机滤膜，进行GC-MS分析。

## 2 结果与讨论

### 2.1 材料表征

采用扫描电子显微镜（SEM）对合成的CS和CS@ZIF-8进行形貌表征。图2a显示CS表面不平坦且粗糙，可以为ZIF-8提供生长位点。图2b 显示ZIF-8纳米颗粒均匀生长在CS表面。图2c 显示制备的CS@ZIF-8复合微球直径在1.5 mm左右。

**图2:**
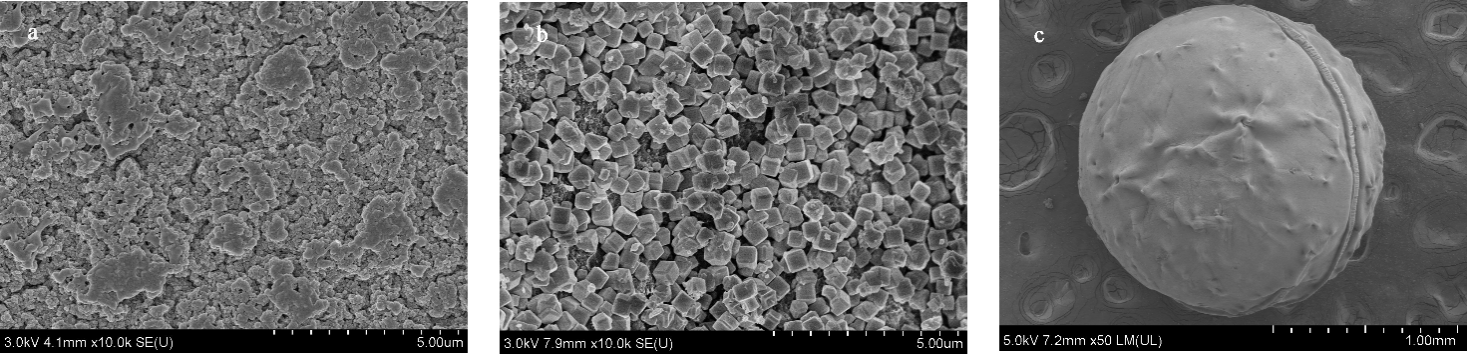
（a）CS和（b，c）CS@ZIF-8的扫描电子显微镜图

通过X射线衍射（XRD）对CS、ZIF-8和CS@ZIF-8的晶体结构进行表征，如图3a所示，ZIF-8的曲线在7.3°、10.4°、12.7°、14.7°、16.5°和18.0°处出现衍射峰，且在7.3°处观察到强而尖锐的峰，这与文献［^29^］的报道一致。CS的曲线在20.1°处呈现宽峰，表明CS是无定形的。在CS@ZIF-8中呈现ZIF-8相关衍射峰，而CS的特征峰的强度降低，这是因为ZIF-8比CS结晶度高。

**图3:**
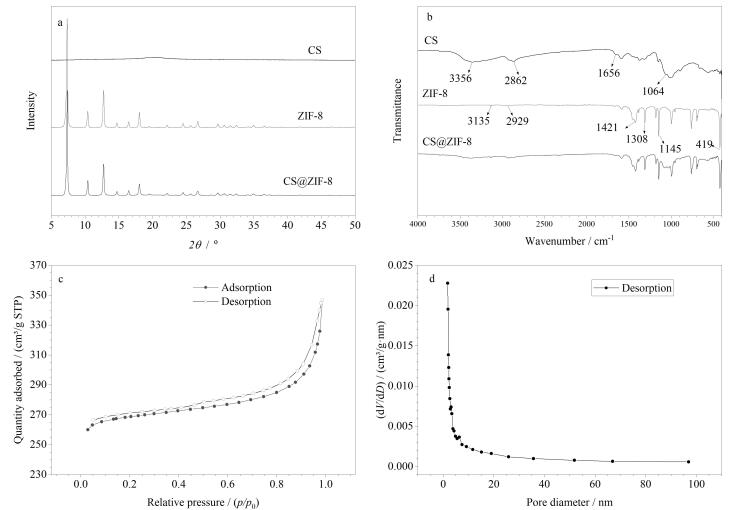
（a）CS、ZIF-8和CS@ZIF-8的X射线衍射图，（b）CS、ZIF-8和CS@ZIF-8的傅里叶红外光谱图，（c）CS@ZIF-8的氮气吸附-脱附等温线和（d）CS@ZIF-8的孔径分布图

图3b为CS、CS@ZIF-8和ZIF-8的傅里叶红外光谱（FT-IR）图。CS在3 356 cm^-1^附近的宽峰是-OH和-NH的伸缩振动峰重叠而增宽的多重吸收峰，在2 862 cm^-1^处的峰对应于C-H的伸缩振动，在1 656 cm^-1^处的峰表示C=O伸缩振动峰，1 064 cm^-1^处的峰对应于C-O-C弯曲振动^［30］^。ZIF-8在3 135 cm^-1^和2 929 cm^-1^处的吸收峰源自C-H伸缩振动，1 421 cm^-1^处出现的特征峰为C=N伸缩振动峰，1 308 cm^-1^和1 145 cm^-1^处的特征峰为C-N伸缩振动峰，419 cm^-1^处的特征峰为Zn-N伸缩振动峰^［31］^。在CS@ZIF-8中观察到CS和ZIF-8的特征峰，表明ZIF-8颗粒成功负载在CS上。

图3c和3d分别显示了CS@ZIF-8复合微球的氮气吸附-脱附等温线和孔径分布。该等温线属于Ⅳ型等温线，BET比表面积为785.74 m²/g，平均孔径为2.70 nm，孔体积为0.53 cm³/g，表明材料存在介孔结构，且具有较高的比表面积，可提供更多的吸附位点。

### 2.2 DSPE条件优化

对影响萃取效率的重要因素，包括 CS@ZIF-8吸附剂用量、离子强度、萃取时间、解吸时间以及解吸溶剂进行优化。优化实验均在100 μg/L水平下进行，每组实验平行测定3次。

#### 2.2.1 CS@ZIF-8吸附剂用量

本实验考察了CS@ZIF-8用量（10~30 mg）对萃取效率的影响。如图4a所示，当CS@ZIF-8用量增加到20 mg时，PCBs的回收率明显提高，进一步增加吸附剂用量回收率不再提升。因此，选择20 mg CS@ZIF-8吸附剂进行后续实验。

**图4:**
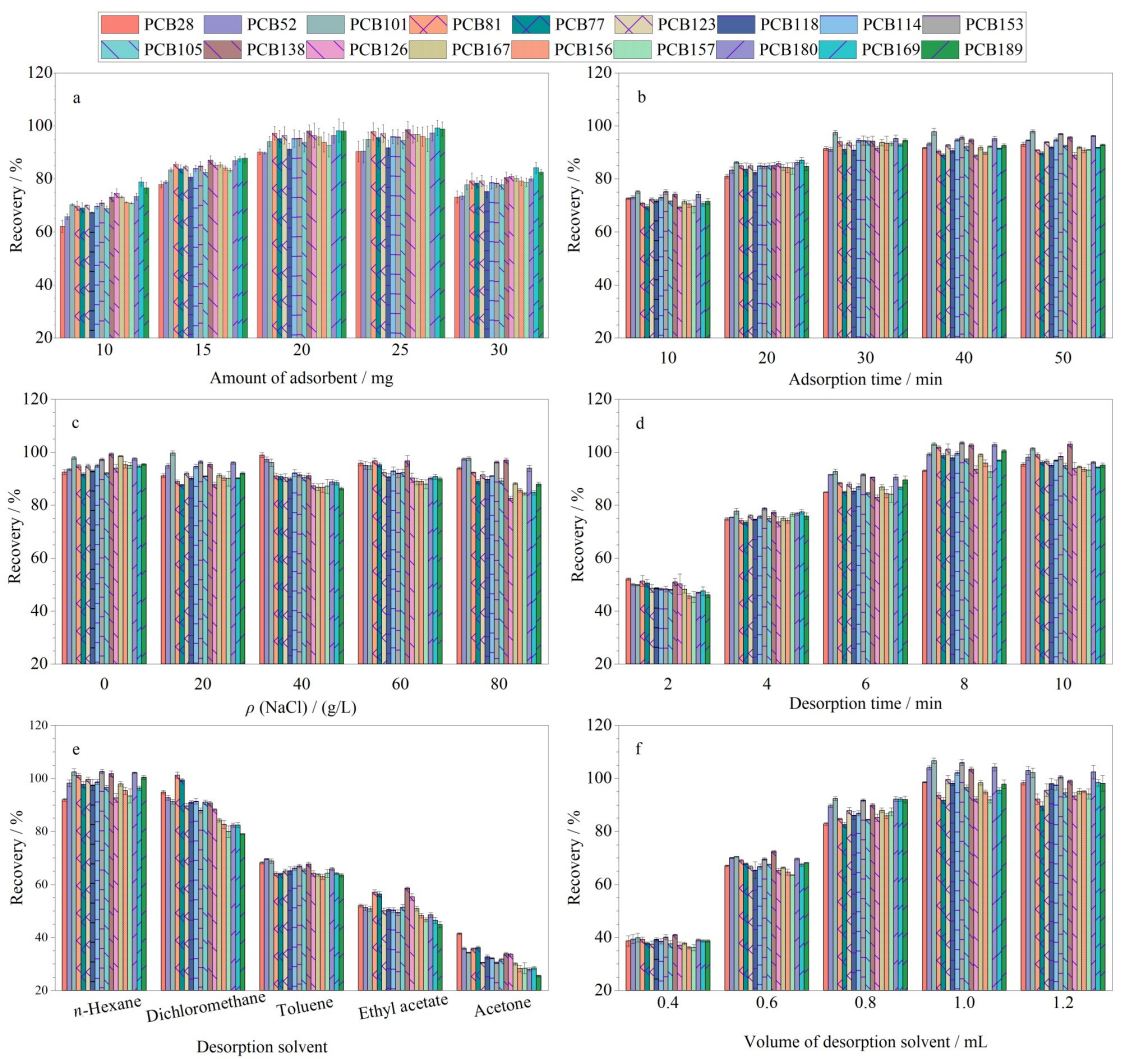
（a）吸附剂用量、（b）吸附时间、（c）离子强度、（d）解吸时间、（e）解吸溶剂类型和（f）解吸溶剂体积对萃取效率的影响（*n*=3）

#### 2.2.2 萃取时间

考察了萃取时间（10~50 min）对萃取效率的影响。如图4b所示，当萃取时间从10 min增加到30 min时，PCBs的回收率逐渐增大，在30 min时达到最大值。随着萃取时间的进一步延长，回收率没有明显变化。因此，选择30 min进行后续实验。

#### 2.2.3 离子强度

如图4c所示，通过添加 0~80 g/L的 NaCl 来考察离子强度对萃取效率的影响，可以看出不同质量浓度NaCl的加入对萃取效率没有显著影响，表明CS@ZIF-8吸附剂与PCBs之间的相互作用不受离子强度的影响。因此，选择不添加NaCl。

#### 2.2.4 解吸时间

考察了解吸时间（2~10 min）对解吸效率的影响。如图4d所示，从2 min到8 min目标物的回收率逐渐提高，继续延长解吸时间，回收率基本保持不变，因此选取8 min为最佳解吸时间。

#### 2.2.5 解吸溶剂类型

PCBs具有较高的疏水性，因此考察了解吸溶剂对解吸效率的影响。选择5种有机溶剂来评价不同解吸溶剂的解吸效果，结果如图4e所示，正己烷解吸效果最好，二氯甲烷次之，丙酮最差。因此，选择正己烷为最佳解吸溶剂。

#### 2.2.6 解吸溶剂体积

本实验考察了解吸溶剂体积（0.4~1.2 mL）对解吸效率的影响。如图4f所示，解吸溶剂体积从0.4 mL增加到1.0 mL时，回收率逐渐增大，在1.0 mL时达到最佳值。因此，选择1.0 mL正己烷进行进一步实验。

### 2.3 萃取性能考察

比较了CS和CS@ZIF-8的吸附性能，如图5所示，CS@ZIF-8的萃取效率明显高于CS。这归因于CS@ZIF-8具有*π*电子共轭体系、疏水性以及大的比表面积，能够增强其对PCBs的萃取效果。

**图5:**
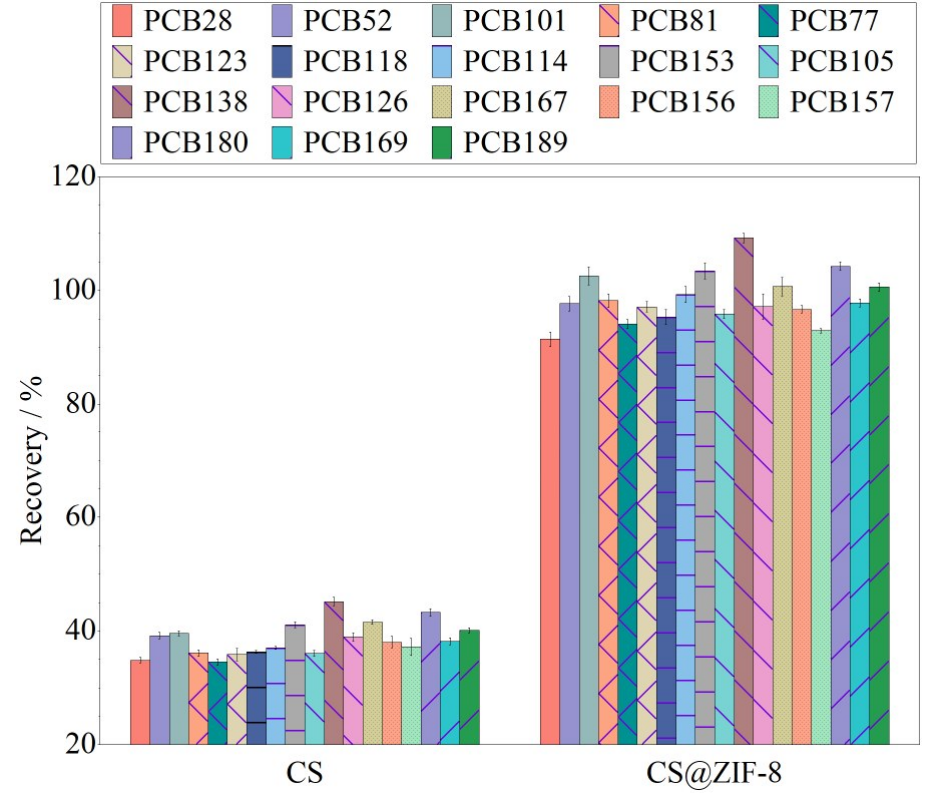
不同吸附剂对萃取效率的影响（*n*=3）

评估CS@ZIF-8复合微球的重复利用性。从图6可以看出，在重复使用4次后，CS@ZIF-8复合微球的萃取效率仍能达到70%以上，表明其具有良好的稳定性，可至少使用4次。

**图6:**
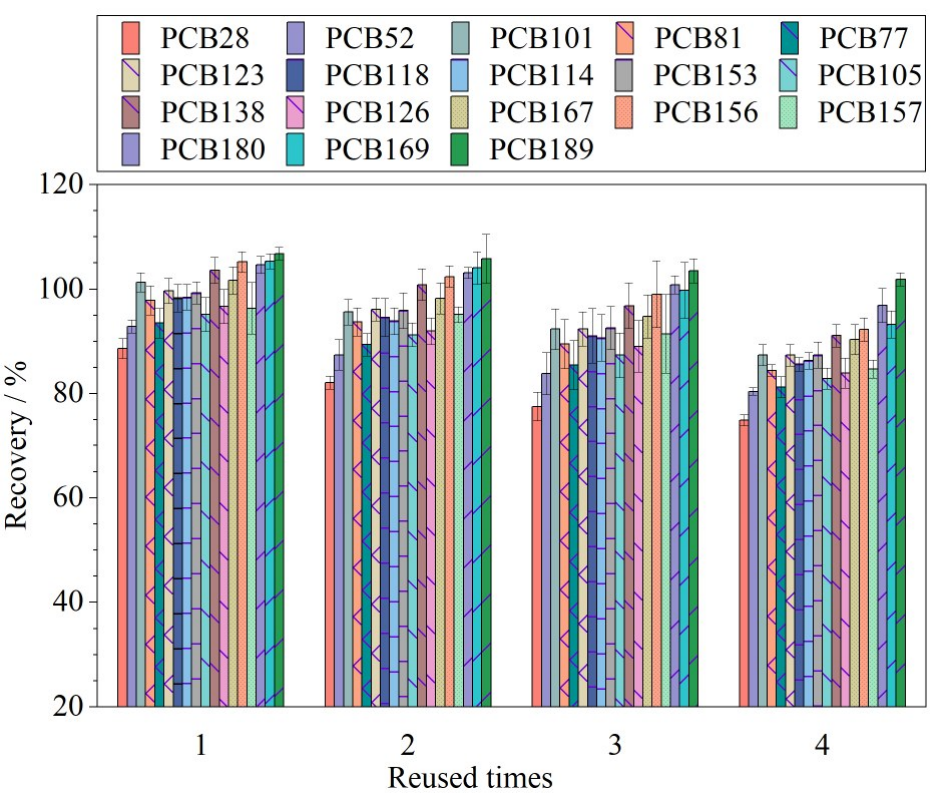
CS@ZIF-8的重复利用次数对萃取效率的影响（*n*=3）

### 2.4 基质效应评价

将空白牛奶样品按1.5节处理后得到基质空白溶液，同时用基质空白溶液和溶剂（正己烷）配制基质标准曲线和溶剂标准曲线，通过基质标准曲线斜率和溶剂标准曲线斜率比值评价基质效应强弱。当斜率比<1时，为基质抑制效应；当斜率比>1时，为基质增强效应；当0.8≤斜率比≤1.2时，通常认为是弱基质效应，可以忽略不计^［32］^。结果表明，18种PCBs的基质效应在1.2~1.6范围内，为基质增强效应，故采用基质空白溶液配制标准曲线。

### 2.5 方法学考察

配制18种PCBs的系列标准溶液，在最佳条件下采用 GC-MS进行分析，以PCBs质量浓度为横坐标，以对应的峰面积为纵坐标，绘制标准曲线。如表2所示，本方法在1~200 μg/L范围内，线性关系良好，相关系数（*r* ^2^）>0.999。方法的检出限（*S/N*=3）为0.06~0.24 μg/L，定量限（*S/N*=10）为0.19~0.79 μg/L。采用加标样品（100 µg/L）评估日内、日间和3批次材料间的精密度。18种PCBs的日内精密度为2.5%~5.3%，日间精密度为4.3%~5.9%，3批次材料间精密度为4.9%~9.7%，结果表明本方法具有良好的稳定性和重复性。

18种PCBs混合标准溶液（100 μg/L）的总离子流色谱图见图7。

**图7:**
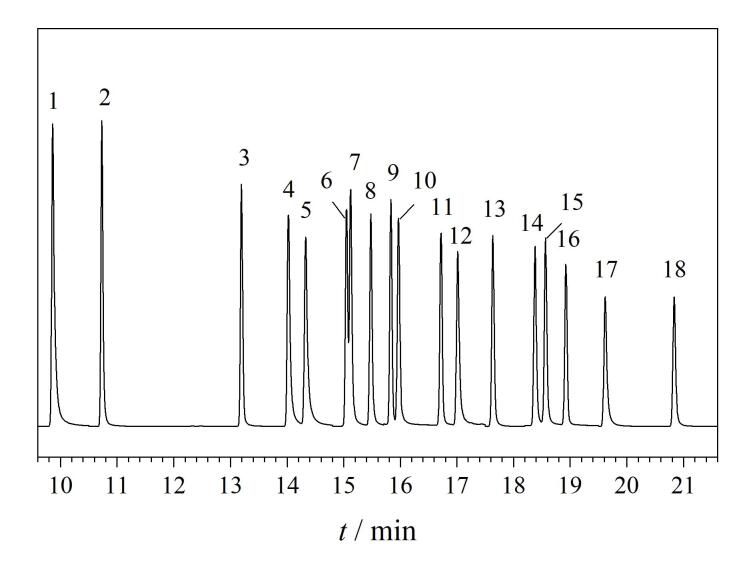
18种PCBs混合标准溶液（100 **μ**g/L）的总离子流色谱图

**表 2:** 18种PCBs的线性范围、相关系数、检出限、定量限及精密度

Analyte	Linear range/（μg/L）	*r* ^2^	LOD/ （μg/L）	LOQ/ （μg/L）	Precisions		
					Intra-day/%（*n*=6）	Inter-day/%（*n*=6）	Batch-to-batch/ % （*n*=3）
PCB28	1‒200	0.9996	0.10	0.32	5.3	4.4	5.2
PCB52	1‒200	0.9997	0.12	0.40	4.7	4.3	5.7
PCB101	1‒200	0.9997	0.06	0.19	3.6	4.7	6.0
PCB81	1‒200	0.9995	0.10	0.32	3.4	5.2	6.4
PCB77	1‒200	0.9993	0.11	0.36	3.6	5.4	7.7
PCB123	1‒200	0.9997	0.14	0.48	3.5	4.8	6.3
PCB118	1‒200	0.9994	0.14	0.45	4.0	4.9	4.9
PCB114	1‒200	0.9994	0.15	0.50	3.6	4.7	5.6
PCB153	1‒200	0.9993	0.17	0.56	3.6	4.6	5.3
PCB105	1‒200	0.9993	0.16	0.53	3.4	4.8	6.3
PCB138	1‒200	0.9995	0.19	0.62	3.0	4.4	5.7
PCB126	1‒200	0.9994	0.18	0.59	2.5	5.6	7.2
PCB167	1‒200	0.9994	0.16	0.52	2.9	4.9	5.8
PCB156	1‒200	0.9994	0.18	0.59	2.5	4.8	5.8
PCB157	1‒200	0.9999	0.19	0.62	3.0	5.3	7.1
PCB180	1‒200	0.9995	0.08	0.28	3.2	4.7	5.7
PCB169	1‒200	0.9993	0.24	0.79	3.1	5.9	9.7
PCB189	1‒200	0.9993	0.18	0.59	2.9	5.2	7.3

### 2.6 实际样品分析

选择市售的全脂牛奶、脱脂牛奶作为实际样品进行分析，在两种牛奶中均未检测出PCBs。向样品中分别添加3个水平（5、20、100 μg/L）的PCBs标准溶液进行加标回收试验，每个浓度平行测定3次。结果见表3，平均加标回收率为84.8%~114.3%，相对标准偏差为0.8%~7.0%。表明本方法适用于检测牛奶中的痕量PCBs。

**表 3:** 18种PCBs在牛奶中的加标回收率和相对标准偏差 （*n*=3）

Analyte	Spiked recoveries （RSDs）/%						
	Whole milk			Skimmed milk			
	5 µg/L	20 µg/L	100 µg/L	5 µg/L	20 µg/L	100 µg/L	
PCB28	84.8 （2.5）	85.0 （5.8）	88.3 （3.6）	87.5 （1.4）	85.1 （3.4）	89.4 （3.7）	
PCB52	89.9 （3.7）	96.3 （4.3）	91.2 （3.6）	93.6 （2.0）	98.9 （5.7）	92.2 （4.3）	
PCB101	92.5 （3.2）	102.1 （2.6）	93.5 （4.3）	96.2 （7.0）	105.1 （5.5）	94.4 （4.3）	
PCB81	90.3 （1.6）	88.7 （5.4）	89.2 （4.6）	93.5 （1.7）	86.5 （6.4）	89.9 （2.0）	
PCB77	90.6 （0.8）	85.3 （4.7）	89.8 （4.9）	94.5 （2.8）	85.0 （2.0）	90.4 （1.8）	
PCB123	97.0 （4.6）	97.2 （1.3）	90.2 （5.6）	95.5 （3.8）	98.0 （4.6）	91.0 （3.7）	
PCB118	93.0 （3.3）	96.4 （5.0）	89.3 （3.5）	92.3 （4.7）	97.0 （5.1）	90.9 （3.1）	
PCB114	100.9 （4.4）	97.1 （2.6）	89.9 （4.4）	98.8 （6.7）	97.8 （4.9）	90.7 （3.1）	
PCB153	110.3 （4.6）	107.8 （3.2）	92.5 （4.4）	107.2 （6.4）	110.9 （5.7）	93.3 （4.2）	
PCB105	105.8 （3.6）	96.4 （5.4）	89.9 （4.3）	100.7 （2.9）	94.0 （4.9）	90.8 （3.2）	
PCB138	108.2 （5.8）	103.7 （3.2）	93.3 （4.0）	105.4 （5.1）	106.5 （6.2）	93.9 （4.6）	
PCB126	95.7 （4.8）	89.4 （6.9）	92.1 （6.5）	92.8 （4.6）	88.4 （6.0）	92.9 （1.6）	
PCB167	106.7 （4.2）	103.0 （3.7）	94.8 （4.9）	102.8 （6.0）	105.8 （5.8）	95.4 （2.4）	
PCB156	110.9 （4.5）	102.4 （3.6）	94.0 （5.7）	104.9 （5.7）	103.4 （5.9）	94.2 （1.6）	
PCB157	103.7 （5.3）	103.5 （4.0）	97.0 （6.3）	98.1 （7.0）	101.1 （5.5）	96.8 （3.1）	
PCB180	111.9 （1.6）	114.3 （2.1）	99.1 （4.0）	109.5 （4.7）	112.1 （1.7）	100.0 （3.1）	
PCB169	102.5 （4.5）	99.9 （4.5）	99.5 （5.7）	99.2 （5.2）	100.1 （6.9）	100.8 （2.1）	
PCB189	111.7 （2.4）	113.3 （3.6）	102.2 （4.1）	108.2 （5.3）	111.1 （2.8）	103.8 （2.1）	

### 2.7 与其他方法的比较

将本方法的实验方法、吸附剂类型、样品类型、萃取时间、检出限、回收率、目标物数量和可重复次数与已发表文献中检测PCBs的方法进行比较（见表4）。本实验的材料制备完成后可直接用于萃取吸附PCBs，不用在制备过程中引入磁性离子，也无需对材料进行涂覆或填充。本方法萃取时间短，可用于复杂样品基质（如牛奶样品）中18种PCBs的同时检测，且检出限和回收率水平相当或更优。综合比较，本方法具有操作简便、萃取时间短、检出限低和准确度高等优点。

**表 4:** 该方法与其他文献方法的比较

Method	Adsorbent	Sample	Extraction time/min	LOD/ （μg/L）	Recoveries/%	Type of PCBs	Reusability	Ref.
MSPE/GC-MS	Fe_3_O_4_@SiO_2_@CTS	water	120	0.02-0.15	90.3-107.0	3	10	［^33^］
MEPS/GC-MS	C_18_	bovine serum	-	0.06-0.53	60.0-91.4	7	-	［^34^］
SBSE/HPLC-DAD	PAF-47/PDMS	water	60	0.04-0.07	81.0-113.0	5	40	［^35^］
SPME/GC-MS	MoS_2_/RGO	milk	40	0.05-0.09	91.4-97.4	7	80	［^36^］
MSPE/GC-MS	Fe_3_O_4_@SiO_2_@C_18_	milk	6	0.10	79.0-116.0	6	3	［^37^］
DSPE-GC-MS	CS@ZIF-8	milk	30	0.06-0.24	84.8-114.3	18	4	this work

MSPE： magnetic solid-phase extraction； MEPS： microextraction by packed sorbent； SBSE： stir bar sorptive extraction； -： no relevant information in the literature.

## 3 结论

本研究采用简单、环保的方法合成了一种比表面积大、结构稳定的CS@ZIF-8复合微球，将CS@ZIF-8用于DSPE，建立了DSPE-GC-MS检测牛奶中18种PCBs的方法。CS@ZIF-8复合微球通过*π*-*π*堆积和疏水作用增强了对PCBs的吸附效果。该方法操作简便，萃取时间短，灵敏度和准确度高，为牛奶中PCBs的高效检测提供了新思路。
